# Supplementary material for: Quorum sensing via dynamic cytokine signaling comprehensively explains divergent patterns of effector choice among helper T cells
Source: PLoS Comput Biol. 2020 Jul 30;16(7):e1008051. doi: 10.1371/journal.pcbi.1008051 (PMC7392205; doi:10.1371/journal.pcbi.1008051)
Supplement: S1 Text — (DOCX) [file pcbi.1008051.s001.docx]

**SUPPORTING TEXT 1: Model Structure, Units, and Incorporation of Cell Density**

Our model has four state variables: expression levels of the transcription factors T-bet (TF_1_) and GATA3 (TF_2_), and expression levels of the cytokines IFNγ (CY_1_) and IL-4 (CY_2_). These molecules influence each other’s expression via the interactions depicted in Fig 1. As is standard, we use Hill functions to describe each of these interactions, and exponential decay to describe the spontaneous loss of each molecule. As in [1,2], each equation is constructed as follows. First, Hill functions that represent each activator of a molecule’s expression are summed. Then, Hill functions that represent inhibitors of a molecule’s expression are multiplied to the specific activation term that they inhibit. Finally, a spontaneous decay term is subtracted from the end of the equation. While this method of constructing such equations is common in the literature, inclusion of equations representing cytokines as dynamic state variables is new.

Regarding units, we defined TF_1_ and TF_2_ as the number of mRNA transcripts encoding T-bet and GATA3, respectively, per cell, to allow direct comparison of our model output with the empirical smFISH measurements of these transcript levels reported by Fang et al [3]. Fang et al report that mRNA transcript count was strongly correlated with the corresponding transcription factor protein expression, suggesting that protein translation occurs on a timescale much faster than the processes we model here and can safely be ignored. Thus, while our state variables TF_1_ and TF_2_ represent mRNA transcript counts per cell, they can also be interpreted as the functionally equivalent protein expression level per cell.

Because TF_1_ and TF_2_ are measured on a per-cell basis, we sought to define cytokines IFNγ (CY_1_) and IL-4 (CY_2_) in a parallel way. Secreted cytokines are typically measured by concentration (e.g. ng/mL, μM, etc.), so we defined our measure of concentration to be the number of cytokine molecules per cell-volume of extracellular space. A “cell-volume” of extracellular space is a volume of extracellular space equivalent to the volume of one Th cell. Given the molecular weight of the cytokines of interests (both roughly 15kDa) and the volume a Th cell (roughly 180μm^3^), the number of cytokine molecules per cell-volume of extracellular space is easily converted to ng/mL (or other measures of concentration), and vice-versa.

Cell density controls the ratio of the volume occupied by cells to the total volume including extracellular space (i.e. packing efficiency).

$$packing efficiency=cell density*cellular volume$$

Packing efficiency is then easily converted into the number of cell-volumes of extracellular space per Th cell (*cvpc*).

$$cvpc= \frac{1-packing efficiency}{packing efficiency}$$

For example, at 10^6^ Th cells/mL, for each Th cell there are ~5567 cell-volumes of extracellular space (*cvpc* = 5567). At 10^9^ Th cells/mL, for each Th cell there are ~4.6 cell-volumes of extracellular space (*cvpc* = 4.6).

Of all the biochemical parameters in the model, only those which describe rates of release or removal of cytokines by cells to/from the extracellular environment depend on *cvpc* and therefore on cell density. These parameters are $a_{1,2}$ and $d_{CY1,2}$. $a_{1,2}$ is the secretion rate of cytokines into the extracellular environment in terms of concentration, which depends on the raw secretion rate of cytokines by Th cells and the diffusion of those cytokines into the available extracellular space. Diffusion of cytokines to a uniform distribution across the extracellular environment happens fast enough *in vitro* that it can be considered instantaneous [4]. Therefore, the cytokine secretion rate in terms of concentration is given by:

$$a_{1,2}=\frac{raw secretion rate}{cvpc} = \frac{72000 molecules/h}{cvpc}$$

See S1 Table for justification of the chosen parameter values.

$d_{CY1,2}$ is the removal rate of cytokines from the extracellular environment in terms of concentration. Like $a_{1,2}$, $d_{CY1,2}$ depends on the raw consumption rate of cytokines by Th cells and the distribution of those cytokines across the available extracellular space. However, cytokines in the extracellular environment also have a substantial rate of free decay. Therefore, the cytokine removal rate in terms of concentration is given by:

$$d_{CY1,2}=\frac{raw consumption rate}{cvpc}+free decay rate= \frac{0.8/h}{cvpc}+0.015/h$$

See S1 Table for justification of the chosen parameter values.

Note that $a_{1,2}$ and $d_{CY1,2}$ do not scale equivalently with cell density. Over the range of cell densities studied here, $a_{1,2}$ increases faster with cell density than does $d_{CY1,2}$ (S1 Fig). This means that at higher cell densities, cytokines in the extracellular environment accumulate to higher concentrations and turn over more dynamically than at lower cell densities.

REFERENCES

1. Antebi YE, Reich-Zeliger S, Hart Y, Mayo A, Eizenberg I, Rimer J, et al. Mapping differentiation under mixed culture conditions reveals a tunable continuum of T cell fates. PLoS Biol. 2013;11(7):e1001616. <https://doi.org/10.1371/journal.pbio.1001616>. PMID: 23935451.
2. Yates A, Callard R, Stark J. Combining cytokine signaling with T-bet and GATA-3 regulation in Th1 and Th2 differentiation: a model for cellular decision-making. J. Theor Biol. 2004; 231(2):181-96. <https://doi.org/10.1016/j.jtbi.2004.06.013>. PMID: 15380383.
3. Fang M, Xie H, Dougan SK, Ploegh H, van Oudenaarden A. Stochastic cytokine expression induces mixed T helper cell states. PLoS Biol. 2013;11(7):e1001618. <https://doi.org/10.1371/journal.pbio.1001618>. PMID: 23935453.
4. Altan-Bonnet G, Mukherjee R. Cytokine-mediated communication: a quantitative appraisal of immune complexity. Nat Rev Immunol. 2019;19(4):205-17. <https://doi.org/10.1038/s41577-019-0131-x>. PMID: 30770905.
