# Supplementary material for: Quorum sensing via dynamic cytokine signaling comprehensively explains divergent patterns of effector choice among helper T cells
Source: PLoS Comput Biol. 2020 Jul 30;16(7):e1008051. doi: 10.1371/journal.pcbi.1008051 (PMC7392205; doi:10.1371/journal.pcbi.1008051)
Supplement: S2 Text — (DOCX) [file pcbi.1008051.s002.docx]

**SUPPORTING TEXT 2: Sensitivity Analyses**

While our parameter assignments are grounded in immunological literature (S1 Table), we performed sensitivity analyses to ensure that the model behavior does not depend on this unique parameter set. By assuming the system is symmetric and non-dimensionalizing the equations, we reduced the number of parameters to 10 (S4 Text). Relaxing the assumption of symmetry introduces an 11^th^ parameter, denoted “Asym.” Two of these 11 parameters – G and L – depend on cell density (S1 Text and S4 Text). Therefore, we had two “points-of-interest” in 11-dimensional parameter space: one representing low cell density (~10^6^ cells/mL) and one representing high cell density (~10^9^ cells/mL). Our sensitivity analyses were conducted on these 11 parameters, centered at these two points-of-interest (between which only G and L differ).

First, we wanted to be sure that no parameter exerted excessive control over the position(s) of the system’s equilibria. At each of the two parameter points-of-interest, we allowed all 11 parameters to independently vary uniformly +/- 10% from their assigned values, and then we sampled 1000 points from this region in parameter space. The number of stable equilibria was one for all 1000 sampled parameter points near the low-density point-of-interest, and it was two for all 1000 sampled parameter points near the high-density point-of-interest. For each point-of-interest, we then fit a first-order linear regression model using the equilibrium position as the dependent variable, and the values of all 11 parameters as the independent variables (after standardizing all 12 variables). For the low-density and high-density points-of-interest, respectively, no parameter had an effect size greater than 5% or 6% in magnitude, despite varying up to +/- 10% (S3a Fig and S3b Fig). This indicates that no parameter excessively influences the positions of the system’s equilibria.

Nonetheless, because parameters D and F appeared most influential at both points-of-interest, we wanted to verify that these parameters did not have locally non-linear first-order or interaction effects on the position of the equilibrium that our linear model could not detect. Fixing the other parameters at their originally assigned values, we systematically varied D and F +/- 5% and calculated the position of the equilibria as a function of these two parameters. Indeed, the local influences of both parameters are essentially linear and non-interacting (S3c Fig and S3d Fig). Together, these analyses indicate that qualitative model behavior does not change as parameters deviate from their originally assigned values, and that quantitative model behavior only changes smoothly and slightly.

Finally, we investigated how drastically parameter values must deviate from their originally assigned values if model behavior were to change qualitatively. For each of the two parameter points-of-interest, we repeated our uniform sampling of 1000 points from a region of parameter space, allowing parameter values to vary +/- 20% from their originally assigned values, +/- 30%, and so on up to +/- 90%. Across both points-of-interest and all levels of parameter variation, the majority of samples returned the same number of stable equilibria as calculated for the originally assigned parameter values (S4a Fig and S4b Fig). This shows that our chosen parameter values at both points-of-interest are as representative as possible of their broader surrounding regions in parameter space. However, even as little as +/- 20% variation in parameter values for the low-density point-of-interest permits some new model behavior, as shown by the rare appearance of numbers of stable equilibria greater than one (S4a Fig). Meanwhile, with up to +/- 50% variation in parameter values for the high-density point-of-interest, no new model behavior was observed (S4b Fig). Given that the low-density point-of-interest represents *in vitro* conditions while the high-density point-of-interest represents *in vivo* conditions, this is sensible. The more biochemical parameters can vary without changing the qualitative behavior of their system, the more robust that system is to perturbations from environmental fluctuations and genetic mutations [1]. If evolution is expected to favor such robustness, then robustness against parameter variation should be observed in conditions under which the biochemical system evolved, but not necessarily under other conditions. Therefore, it is sensible that we observe more robustness against parameter variation under *in vivo* compared to *in vitro* conditions. Overall, these analyses suggest that our model behavior for the assigned parameter set is representative of the broader surrounding region of parameter space.

REFERENCES

1. Zamora-Sillero E, Hafner M, Ibig A, Stelling J, Wagner A. Efficient characterization of high-dimensional parameter spaces for systems biology. BMC Syst Biol. 2011;5:142. <https://doi.org/10.1186/1752-0509-5-142>. PMID: 21920040.
