# Supplementary material for: Quorum sensing via dynamic cytokine signaling comprehensively explains divergent patterns of effector choice among helper T cells
Source: PLoS Comput Biol. 2020 Jul 30;16(7):e1008051. doi: 10.1371/journal.pcbi.1008051 (PMC7392205; doi:10.1371/journal.pcbi.1008051)
Supplement: S3 Text — (DOCX) [file pcbi.1008051.s003.docx]

**SUPPORTING TEXT 3: Extension to Stochastic Differential Equations**

In order to study the full distribution of molecular expression across cells that stochastically vary in their expression of each molecule, we extended each equation in our model to a stochastic differential equation (SDE), as follows:

$$\mathrm{Eq}n 1 ⅆ\boldsymbol{TF}_{1}=\left[ \left( b+\frac{p_{1}\boldsymbol{TF}_{1}^{h_{p}}}{P_{1}^{h_{p}}+\boldsymbol{TF}_{1}^{h_{p}}} \right)\left( \frac{X_{2}^{h_{x}}}{X_{2}^{h_{x}}+\boldsymbol{TF}_{2}^{h_{x}}} \right)+\left( \frac{s_{1}{\boldsymbol{CY}_{1}}^{h_{s}}}{S_{1}^{h_{s}}+{\boldsymbol{CY}_{1}}^{h_{s}}} \right)\left( \frac{Z_{2}^{h_{z}}}{Z_{2}^{h_{z}}+\boldsymbol{CY}_{2}^{h_{z}}} \right)-d_{TF1}\boldsymbol{TF}_{1} \right]dt+ \left[ n_{TF}\boldsymbol{TF}_{\boldsymbol{1}} \right]{dW}_{TF1}$$

$$\mathrm{Eq}n 2 ⅆ\boldsymbol{TF}_{2}=\left[ \left( b+\frac{p_{2}\boldsymbol{TF}_{2}^{h_{p}}}{P_{2}^{h_{p}}+\boldsymbol{TF}_{2}^{h_{p}}} \right)\left( \frac{X_{1}^{h_{x}}}{X_{1}^{h_{x}}+\boldsymbol{TF}_{1}^{h_{x}}} \right) +\left( \frac{s_{2}{\boldsymbol{CY}_{2}}^{h_{s}}}{S_{2}^{h_{s}}+{\boldsymbol{CY}_{2}}^{h_{s}}} \right)\left( \frac{Z_{1}^{h_{z}}}{Z_{1}^{h_{z}}+\boldsymbol{CY}_{1}^{h_{z}}} \right)-d_{TF2}\boldsymbol{TF}_{2} \right]dt+ \left[ n_{TF}\boldsymbol{TF}_{\boldsymbol{2}} \right]{dW}_{TF2}$$

$$\mathrm{Eq}n 3 ⅆ\boldsymbol{CY}_{1}=\left[ \left( \frac{a_{1}\boldsymbol{TF}_{1}^{h_{a}}}{A_{1}^{h_{a}}+\boldsymbol{TF}_{1}^{h_{a}}} \right)\left( \frac{R_{2}^{h_{r}}}{R_{2}^{h_{r}}+\boldsymbol{TF}_{2}^{h_{r}}} \right)\left( \frac{U_{2}^{h_{u}}}{U_{2}^{h_{u}}+\boldsymbol{CY}_{2}^{h_{u}}} \right)-d_{CY1}\boldsymbol{CY}_{1} \right]dt+ \left[ n_{CY}\boldsymbol{CY}_{\boldsymbol{1}} \right]{dW}_{CY1}$$

$$\mathrm{Eq}n 4 ⅆ\boldsymbol{CY}_{2}=\left[ \left( \frac{a_{2}\boldsymbol{TF}_{2}^{h_{a}}}{A_{2}^{h_{a}}+\boldsymbol{TF}_{2}^{h_{a}}} \right)\left( \frac{R_{1}^{h_{r}}}{R_{1}^{h_{r}}+\boldsymbol{TF}_{1}^{h_{r}}} \right)\left( \frac{U_{1}^{h_{u}}}{U_{1}^{h_{u}}+\boldsymbol{CY}_{1}^{h_{u}}} \right)-d_{\mathrm{CY}2}\boldsymbol{CY}_{2} \right]dt+ \left[ n_{CY}\boldsymbol{CY}_{\boldsymbol{2}} \right]{dW}_{CY2}$$

The dW terms are the differentials of independent Brownian motion processes, i.e. independent Wiener processes. The magnitude of cell-to-cell variability is given by the noise coefficient n, which can differ between the transcription factor and the cytokine equations. The structure of the stochastic term, noise coefficient * state variable * dW, represents a geometric Brownian motion process, which causes the stochastic fluctuations in each state variable to be lognormal. This is convenient in our setting because the cell-to-cell variabilities of all four molecules these equations represent – T-bet, GATA3, IFNγ, and IL-4 – have been empirically shown to follow positively-skewed distributions quite close to lognormal [1,2]. While this match in distributions allows us to study biologically realistic stochasticity with a mathematically simple construct, it is a phenomenological implementation that does not represent stochastic fluctuations in specific parameter values propagating through to the state variables. Therefore, the parameter n cannot be interpreted as the fluctuation in any particular biochemical parameter, but rather it is a unitless quantification of the total cell-to-cell variability of each state variable, integrating over all biochemical sources (even those which do not appear in the equations).

Each time the full distribution of molecular expression across cells was required, we ran forward simulations of 100-1000 sample paths of this SDE system. These allowed us to estimate the mean, median, and spread in the expression of each molecule across a population of cells at any time point, wherever required.

**REFERENCES**

1. Fang M, Xie H, Dougan SK, Ploegh H, van Oudenaarden A. Stochastic cytokine expression induces mixed T helper cell states. PLoS Biol. 2013;11(7):e1001618. <https://doi.org/10.1371/journal.pbio.1001618>. PMID: 23935453.
2. Han Q, Bradshaw EM, Nilsson B, Hafler DA, Love JC. Multidimensional analysis of the frequencies and rates of cytokine secretion from single cells by quantitative microengraving. Lab Chip. 2010;10(11):1391-400. <https://doi.org/10.1039/b926849a>. PMID: 20376398.
