# Supplementary material for: Quorum sensing via dynamic cytokine signaling comprehensively explains divergent patterns of effector choice among helper T cells
Source: PLoS Comput Biol. 2020 Jul 30;16(7):e1008051. doi: 10.1371/journal.pcbi.1008051 (PMC7392205; doi:10.1371/journal.pcbi.1008051)
Supplement: S4 Text — (DOCX) [file pcbi.1008051.s004.docx]

**SUPPORTING TEXT 4: Mathematical Analysis of the Model Bifurcation**

To aid in mathematical analysis, we first assume that parameter values are symmetric; that is, parameter_1_ = parameter_2_ for all parameters, so that subscripts can be dropped from all parameters. We then non-dimensionalize the four-ODE system, beginning with the **TF** equations:

$$Eq. 1 \frac{ⅆ\boldsymbol{TF}_{1}}{dt}=\left( b+\frac{{p\boldsymbol{TF}}_{1}^{h_{p}}}{P^{h_{p}}+\boldsymbol{TF}_{1}^{h_{p}}} \right)\left( \frac{X^{h_{x}}}{X^{h_{x}}+\boldsymbol{TF}_{2}^{h_{x}}} \right)+\left( \frac{s{\boldsymbol{CY}_{1}}^{h_{s}}}{S^{h_{s}}+{\boldsymbol{CY}_{1}}^{h_{s}}} \right)\left( \frac{Z^{h_{z}}}{Z^{h_{z}}+\boldsymbol{CY}_{2}^{h_{z}}} \right)-d_{\mathrm{TF}}\boldsymbol{TF}_{1}$$

Introduce a non-dimensional variable $\boldsymbol{N}_{1}=\frac{\boldsymbol{TF}_{1}}{X}$ to rescale **TF**_1_, and symmetrically for **TF**_2_

$$Eq. 2 \frac{d\boldsymbol{N}_{1}}{dt}=\frac{1}{X}\frac{d\boldsymbol{TF}_{1}}{dt}=\frac{1}{X}\left( b+\frac{{p\boldsymbol{N}}_{1}^{h_{p}}}{\left( \frac{p}{X} \right)^{h_{p}}+\boldsymbol{N}_{1}^{h_{p}}} \right)\left( \frac{1}{1+\boldsymbol{N}_{2}^{h_{x}}} \right)+\frac{1}{X}\left( \frac{s{\boldsymbol{CY}_{1}}^{h_{s}}}{S^{h_{s}}+{\boldsymbol{CY}_{1}}^{h_{s}}} \right)\left( \frac{Z^{h_{z}}}{Z^{h_{z}}+\boldsymbol{CY}_{2}^{h_{z}}} \right)-d_{\mathrm{TF}}\boldsymbol{N}_{1}$$

Introduce non-dimensional time $T= \frac{p}{X}t$

$$Eq. 3 \frac{d\boldsymbol{N}_{1}}{dT}=\frac{X}{p}\frac{d\boldsymbol{N}_{1}}{dt}=\left( \frac{b}{p}+\frac{\boldsymbol{N}_{1}^{h_{p}}}{\left( \frac{p}{X} \right)^{h_{p}}+\boldsymbol{N}_{1}^{h_{p}}} \right)\left( \frac{1}{1+\boldsymbol{N}_{2}^{h_{x}}} \right)+\left( \frac{\frac{s}{p}{\boldsymbol{CY}_{1}}^{h_{s}}}{S^{h_{s}}+{\boldsymbol{CY}_{1}}^{h_{s}}} \right)\left( \frac{Z^{h_{z}}}{Z^{h_{z}}+\boldsymbol{CY}_{2}^{h_{z}}} \right)-\frac{d_{\mathrm{TF}}X}{p}\boldsymbol{N}_{1}$$

Introduce a non-dimensional variable $\boldsymbol{M}_{1}=\frac{\boldsymbol{CY}_{1}}{U}$ to rescale **CY**_1_, and symmetrically for **CY**_2_.

$$Eq. 4 \frac{d\boldsymbol{N}_{1}}{dT}=\frac{X}{p}\frac{d\boldsymbol{N}_{1}}{dt}=\left( \frac{b}{p}+\frac{\boldsymbol{N}_{1}^{h_{p}}}{\left( \frac{p}{X} \right)^{h_{p}}+\boldsymbol{N}_{1}^{h_{p}}} \right)\left( \frac{1}{1+\boldsymbol{N}_{2}^{h_{x}}} \right)+\left( \frac{\frac{s}{p}{\boldsymbol{M}_{1}}^{h_{s}}}{\left( \frac{S}{U} \right)^{h_{s}}+{\boldsymbol{M}_{1}}^{h_{s}}} \right)\left( \frac{\left( \frac{Z}{U} \right)^{h_{z}}}{\left( \frac{Z}{U} \right)^{h_{z}}+\boldsymbol{M}_{2}^{h_{z}}} \right)-\frac{d_{\mathrm{TF}}X}{p}\boldsymbol{N}_{1}$$

Rename $B= \frac{b}{p}, H= \frac{p}{X}, F= \frac{s}{p}, E=\frac{S}{U}, W=\frac{Z}{U}, D= \frac{d_{TF}X}{p}$. Note these parameters are dimensionless.

$$Eq. 5 \frac{d\boldsymbol{N}_{1}}{dT}=\left( B+\frac{\boldsymbol{N}_{1}^{h_{p}}}{H^{h_{p}}+\boldsymbol{N}_{1}^{h_{p}}} \right)\left( \frac{1}{1+\boldsymbol{N}_{2}^{h_{x}}} \right)+\left( \frac{F{\boldsymbol{CY}_{1}}^{h_{s}}}{E^{h_{s}}+{\boldsymbol{CY}_{1}}^{h_{s}}} \right)\left( \frac{W^{h_{z}}}{W^{h_{z}}+\boldsymbol{CY}_{2}^{h_{z}}} \right)-D\boldsymbol{N}_{1}$$

The symmetrical equation holds for **N**_2_. Now, move onto the **CY** equations:

$$Eq. 6 \frac{ⅆ\boldsymbol{CY}_{1}}{dt}=\left( \frac{a\boldsymbol{TF}_{1}^{h_{a}}}{A^{h_{a}}+\boldsymbol{TF}_{1}^{h_{a}}} \right)\left( \frac{R^{h_{r}}}{R^{h_{r}}+\boldsymbol{TF}_{2}^{h_{r}}} \right)\left( \frac{U^{h_{u}}}{U^{h_{u}}+\boldsymbol{CY}_{2}^{h_{u}}} \right)-d_{\mathrm{CY}}\boldsymbol{CY}_{1}$$

Recall non-dimensional variables $\boldsymbol{M}_{1}=\frac{\boldsymbol{CY}_{1}}{U}$ and $\boldsymbol{N}_{1}=\frac{\boldsymbol{TF}_{1}}{X}$, and symmetrically for **M**_2_ and **N**_2_.

$$Eq. 7 \frac{d\boldsymbol{M}_{1}}{dt}=\frac{1}{U}\frac{ⅆ\boldsymbol{CY}_{1}}{dt}=\frac{1}{U}\left( \frac{a\boldsymbol{N}_{1}^{h_{a}}}{\left( \frac{A}{X} \right)^{h_{a}}+\boldsymbol{N}_{1}^{h_{a}}} \right)\left( \frac{\left( \frac{R}{X} \right)^{h_{r}}}{\left( \frac{R}{X} \right)^{h_{r}}+\boldsymbol{N}_{2}^{h_{r}}} \right)\left( \frac{1}{1+\boldsymbol{M}_{2}^{h_{u}}} \right)-d_{\mathrm{CY}}\boldsymbol{M}_{1}$$

Recall non-dimensional time $T= \frac{p}{X}t$

$$Eq. 8 \frac{d\boldsymbol{M}_{1}}{dT}=\frac{X}{p}\frac{ⅆ\boldsymbol{M}_{1}}{dt}=\frac{aX}{Up}\left( \frac{\boldsymbol{N}_{1}^{h_{a}}}{\left( \frac{A}{X} \right)^{h_{a}}+\boldsymbol{N}_{1}^{h_{a}}} \right)\left( \frac{\left( \frac{R}{X} \right)^{h_{r}}}{\left( \frac{R}{X} \right)^{h_{r}}+\boldsymbol{N}_{2}^{h_{r}}} \right)\left( \frac{1}{1+\boldsymbol{M}_{2}^{h_{u}}} \right)-\frac{d_{\mathrm{CY}}X}{p}\boldsymbol{M}_{1}$$

Rename $G= \frac{aX}{Up}, V= \frac{A}{X}, Q= \frac{R}{X}, L= \frac{d_{CY}X}{p}$. Note these parameters are dimensionless.

$$Eq. 9 \frac{d\boldsymbol{M}_{1}}{dT}=G\left( \frac{\boldsymbol{N}_{1}^{h_{a}}}{V^{h_{a}}+\boldsymbol{N}_{1}^{h_{a}}} \right)\left( \frac{Q^{h_{r}}}{Q^{h_{r}}+\boldsymbol{N}_{2}^{h_{r}}} \right)\left( \frac{1}{1+\boldsymbol{M}_{2}^{h_{u}}} \right)-L\boldsymbol{M}_{1}$$

The symmetrical equation holds for **M**_2_.

The major bifurcation that we discuss (Fig 4a) occurs because the mixed equilibrium transitions from stable to unstable as cell density increases. To study understand this transition, we must first write the Jacobian, **J**, of the non-dimensionalized four-ODE system:

| $\left( \frac{1}{1+\boldsymbol{N}_{2}^{h_{x}}} \right)\left( \frac{h_{p}H^{h_{p}}\boldsymbol{N}_{1}^{h_{p}-1}}{\left( H^{h_{p}}+\boldsymbol{N}_{1}^{h_{p}} \right)^{2}} \right)-D$ | $-\left( B+\frac{\boldsymbol{N}_{1}^{h_{p}}}{H^{h_{p}}+\boldsymbol{N}_{1}^{h_{p}}} \right)\left( \frac{h_{x}\boldsymbol{N}_{2}^{h_{x}-1}}{\left( 1+\boldsymbol{N}_{2}^{h_{x}} \right)^{2}} \right)$ | $F\left( \frac{W^{h_{z}}}{W^{h_{z}}+\boldsymbol{M}_{2}^{h_{z}}} \right)\left( \frac{h_{s}E^{h_{s}}\boldsymbol{M}_{1}^{h_{s}-1}}{\left( E^{h_{s}}+\boldsymbol{M}_{1}^{h_{s}} \right)^{2}} \right)$ | $-F\left( \frac{\boldsymbol{M}_{1}^{h_{s}}}{E^{h_{s}}+\boldsymbol{M}_{1}^{h_{s}}} \right)\left( \frac{h_{z}W^{h_{z}}\boldsymbol{M}_{2}^{h_{z}-1}}{\left( W^{h_{z}}+\boldsymbol{M}_{2}^{h_{z}} \right)^{2}} \right)$ |
| --- | --- | --- | --- |
| $-\left( B+\frac{\boldsymbol{N}_{2}^{h_{p}}}{H^{h_{p}}+\boldsymbol{N}_{2}^{h_{p}}} \right)\left( \frac{h_{x}\boldsymbol{N}_{1}^{h_{x}-1}}{\left( 1+\boldsymbol{N}_{1}^{h_{x}} \right)^{2}} \right)$ | $\left( \frac{1}{1+\boldsymbol{N}_{1}^{h_{x}}} \right)\left( \frac{h_{p}H^{h_{p}}\boldsymbol{N}_{2}^{h_{p}-1}}{\left( H^{h_{p}}+\boldsymbol{N}_{2}^{h_{p}} \right)^{2}} \right)-D$ | $-F\left( \frac{\boldsymbol{M}_{2}^{h_{s}}}{E^{h_{s}}+\boldsymbol{M}_{2}^{h_{s}}} \right)\left( \frac{h_{z}W^{h_{z}}\boldsymbol{M}_{1}^{h_{z}-1}}{\left( W^{h_{z}}+\boldsymbol{M}_{1}^{h_{z}} \right)^{2}} \right)$ | $F\left( \frac{W^{h_{z}}}{W^{h_{z}}+\boldsymbol{M}_{1}^{h_{z}}} \right)\left( \frac{h_{s}E^{h_{s}}\boldsymbol{M}_{2}^{h_{s}-1}}{\left( E^{h_{s}}+\boldsymbol{M}_{2}^{h_{s}} \right)^{2}} \right)$ |
| $G\left( \frac{Q^{h_{r}}}{Q^{h_{r}}+\boldsymbol{N}_{2}^{h_{r}}} \right)\left( \frac{1}{1+\boldsymbol{M}_{2}^{h_{u}}} \right)$  $\cdot\left( \frac{h_{a}V^{h_{a}}\boldsymbol{N}_{1}^{h_{a}-1}}{\left( V^{h_{a}}+\boldsymbol{N}_{1}^{h_{a}} \right)^{2}} \right)$ | $-G\left( \frac{\boldsymbol{N}_{1}^{h_{a}}}{V^{h_{a}}+\boldsymbol{N}_{1}^{h_{a}}} \right)\left( \frac{1}{1+\boldsymbol{M}_{2}^{h_{u}}} \right)$  $\cdot\left( \frac{h_{r}Q^{h_{r}}\boldsymbol{N}_{2}^{h_{r}-1}}{\left( Q^{h_{r}}+\boldsymbol{N}_{2}^{h_{r}} \right)^{2}} \right)$ | $-L$ | $-G\left( \frac{\boldsymbol{N}_{1}^{h_{a}}}{V^{h_{a}}+\boldsymbol{N}_{1}^{h_{a}}} \right)\left( \frac{Q^{h_{r}}}{Q^{h_{r}}+\boldsymbol{N}_{2}^{h_{r}}} \right)$  $\cdot\left( \frac{h_{u}\boldsymbol{M}_{2}^{h_{u}-1}}{\left( 1+\boldsymbol{M}_{2}^{h_{u}} \right)^{2}} \right)$ |
| $-G\left( \frac{\boldsymbol{N}_{2}^{h_{a}}}{V^{h_{a}}+\boldsymbol{N}_{2}^{h_{a}}} \right)\left( \frac{1}{1+\boldsymbol{M}_{1}^{h_{u}}} \right)$  $\cdot\left( \frac{h_{r}Q^{h_{r}}\boldsymbol{N}_{1}^{h_{r}-1}}{\left( Q^{h_{r}}+\boldsymbol{N}_{1}^{h_{r}} \right)^{2}} \right)$ | $G\left( \frac{Q^{h_{r}}}{Q^{h_{r}}+\boldsymbol{N}_{1}^{h_{r}}} \right)\left( \frac{1}{1+\boldsymbol{M}_{1}^{h_{u}}} \right)$  $\cdot\left( \frac{h_{a}V^{h_{a}}\boldsymbol{N}_{2}^{h_{a}-1}}{\left( V^{h_{a}}+\boldsymbol{N}_{2}^{h_{a}} \right)^{2}} \right)$ | $-G\left( \frac{\boldsymbol{N}_{2}^{h_{a}}}{V^{h_{a}}+\boldsymbol{N}_{2}^{h_{a}}} \right)\left( \frac{Q^{h_{r}}}{Q^{h_{r}}+\boldsymbol{N}_{1}^{h_{r}}} \right)$  $\cdot\left( \frac{h_{u}\boldsymbol{M}_{1}^{h_{u}-1}}{\left( 1+\boldsymbol{M}_{1}^{h_{u}} \right)^{2}} \right)$ | $-L$ |

Because we are concerned with the mixed equilibrium (**N^*^**_1_, **N^*^**_2_, **M^*^**_1_, **M^*^**_2_), where **N^*^**_1_ = **N^*^**_2_ and **M^*^**_1_ = **M^*^**_2_, some of the Jacobian entries will be identical to others. We can name the entries of the Jacobian at the mixed equilibrium, **J***, as follows:

| **a** | **b** | **c** | **d** |  |
| --- | --- | --- | --- | --- |
| **b** | **a** | **d** | **c** |  |
| **e** | **f** | **g** | **h** |  |
| **f** | **e** | **h** | **g** |  |

Note from the generalized Jacobian above that, for the parameter range of interest, the blue entries are always negative, and the red entries are always positive.

Here, we pause to define the tensor product, Ꚛ, of two matrices. Suppose we have two 2x2 matrices: $\alpha= \begin{matrix} \alpha_{1} & \alpha_{2} \\ \alpha_{3} & \alpha_{4} \end{matrix}$ and $\beta= \begin{matrix} \beta_{1} & \beta_{2} \\ \beta_{3} & \beta_{4} \end{matrix}$ . Then the tensor product $\alpha$ Ꚛ$\beta$ is the 4x4 matrix:

| $\alpha_{1}\beta_{1}$ | $\alpha_{1}\beta_{2}$ | $\alpha_{2}\beta_{1}$ | $\alpha_{2}\beta_{2}$ |  |
| --- | --- | --- | --- | --- |
| $\alpha_{1}\beta_{3}$ | $\alpha_{1}\beta_{4}$ | $\alpha_{2}\beta_{3}$ | $\alpha_{2}\beta_{4}$ |  |
| $\alpha_{3}\beta_{1}$ | $\alpha_{3}\beta_{2}$ | $\alpha_{4}\beta_{1}$ | $\alpha_{4}\beta_{2}$ |  |
| $\alpha_{3}\beta_{3}$ | $\alpha_{3}\beta_{4}$ | $\alpha_{4}\beta_{3}$ | $\alpha_{4}\beta_{4}$ |  |

Returning to **J***, the mixed equilibrium will be stable if and only if the real parts of all four eigenvalues of **J*** are negative. Without altering these eigenvalues, we may shuffle rows and columns of **J***. So we swap the two inner columns of **J*** and the two inner rows of **J*** to obtain:

| **a** | **c** | **b** | **d** |  |
| --- | --- | --- | --- | --- |
| **e** | **g** | **f** | **h** |  |
| **b** | **d** | **a** | **c** |  |
| **f** | **h** | **e** | **g** |  |

Note that the upper left and lower right quadrants are now identical, as are the upper right and lower left quadrants. As a result, we can write **J*** as:

**J*** = $\left| \begin{matrix} 1 & 0 \\ 0 & 1 \end{matrix} \right|$ Ꚛ $\left| \begin{matrix} \mathbf{a} & \mathbf{c} \\ \mathbf{e} & \mathbf{g} \end{matrix} \right|$ **+**  $\left| \begin{matrix} 0 & 1 \\ 1 & 0 \end{matrix} \right|$ Ꚛ $\left| \begin{matrix} \mathbf{b} & \mathbf{d} \\ \mathbf{f} & \mathbf{h} \end{matrix} \right|$

Thus, the eigenvalues of **J*** equal those of $\left| \begin{matrix} \mathbf{a} & \mathbf{c} \\ \mathbf{e} & \mathbf{g} \end{matrix} \right|\pm\left| \begin{matrix} \mathbf{b} & \mathbf{d} \\ \mathbf{f} & \mathbf{h} \end{matrix} \right|= \left| \begin{matrix} \mathbf{a}\mathbf{+}\mathbf{b} & \mathbf{c}\mathbf{+}\mathbf{d} \\ \mathbf{e}\mathbf{+}\mathbf{f} & \mathbf{g}\mathbf{+}\mathbf{h} \end{matrix} \right|and \left| \begin{matrix} \mathbf{a}\mathbf{-}\mathbf{b} & \mathbf{c}\mathbf{-}\mathbf{d} \\ \mathbf{e}\mathbf{-}\mathbf{f} & \mathbf{g}\mathbf{-}\mathbf{h} \end{matrix} \right|$ [1,2]. The real parts of all four eigenvalues are negative if and only if the traces of both matrices are negative and the determinants of both matrices are positive. This yields four conditions which must all hold for the mixed equilibrium to be stable:

Cond. 1: **a** + **b** + **g** + **h** < 0

Cond. 2: **a** – **b** + **g** – **h** < 0

Cond. 3 (**a**+**b**)(**g**+**h**) – (**c**+**d**)(**e**+**f**) > 0 🡺 **ag** + **bh** – **ce** – **df** > – (**bg** + **ah** – **cf** – **de**)

Cond. 4 (**a**-**b**)(**g**-**h**) – (**c**-**d**)(**e**-**f**) > 0 🡺 **ag** + **bh** – **ce** – **df** > **bg** + **ah** – **cf** – **de**

Some of these conditions can be ignored. First, because **a**, **b**, **g**, **h** < 0, Cond. 1 is always true. Second, for the parameter range of interest, Cond. 2 is always true. Third, **bg** + **ah** – **cf** – **de** is always positive, so –(**bg** + **ah** – **cf** – **de**) is always negative, so the former expression is always greater than the latter. As a result, Cond. 4 implies Cond. 3. Therefore, only Cond. 4 must be verified for the mixed equilibrium to be stable. Replacing each letter in Cond. 4 with the partial derivative it represents, evaluated at the mixed equilibrium, we obtain:

$$\left( \frac{\partial\frac{d\boldsymbol{N}_{1}}{dt}}{\partial\boldsymbol{N}_{1}}-\frac{\partial\frac{d\boldsymbol{N}_{1}}{dt}}{\partial\boldsymbol{N}_{2}} \right)\left( \frac{\partial\frac{d\boldsymbol{M}_{1}}{dt}}{\partial\boldsymbol{M}_{1}}-\frac{\partial\frac{d\boldsymbol{M}_{1}}{dt}}{\partial\boldsymbol{M}_{2}} \right)> \left( \frac{\partial\frac{d\boldsymbol{N}_{1}}{dt}}{\partial\boldsymbol{M}_{1}}-\frac{\partial\frac{d\boldsymbol{N}_{1}}{dt}}{\partial\boldsymbol{M}_{2}} \right)\left( \frac{\partial\frac{d\boldsymbol{M}_{1}}{dt}}{\partial\boldsymbol{N}_{1}}-\frac{\partial\frac{d\boldsymbol{M}_{1}}{dt}}{\partial\boldsymbol{N}_{2}} \right)$$

On the left, all partial derivatives represent within-scale interactions – transcription factors affecting the expression of transcription factors, and cytokines affecting the expression of cytokines. Moreover, because all partial derivatives are negative, they may counteract one another in subtraction, making the overall product a net effect. Since this product is positive for the parameter range of interest, the net effect is stabilizing.

On the right, all partial derivatives represent cross-scale interactions – cytokines affecting the expression of transcription factors, and transcription factors affecting the expression of cytokines. Moreover, because some partial derivatives are positive and some are negative, they all cooperate toward larger positive values in subtraction, making the overall product a total effect. Since this product is positive for the parameter range of interest, the total effect is destabilizing.

Therefore, this condition can be read as follows: “the mixed equilibrium is stable if and only if the net stabilizing effect of within-scale interactions outweighs the total destabilizing effect of cross-scale interactions.”

**REFERENCES**

1. Othmer HG, Scriven LE. Instability and dynamic pattern in cellular networks. J Theor Biol. 1971;32(3):507-37. <https://doi.org/10.1016/0022-5193(71)90154-8>. PMID: 5571122.
2. Levin SA. Dispersion and population interactions. Am. Nat. 1974;108(960):207-28.
