# Supplementary material for: Quorum sensing via dynamic cytokine signaling comprehensively explains divergent patterns of effector choice among helper T cells
Source: PLoS Comput Biol. 2020 Jul 30;16(7):e1008051. doi: 10.1371/journal.pcbi.1008051 (PMC7392205; doi:10.1371/journal.pcbi.1008051)
Supplement: S1 Table — (DOCX) [file pcbi.1008051.s005.docx]

**SUPPLEMENTAL TABLE**

S1 Table. Parameter interpretations and values.

| **Par.** | **Citation** | **Interpretation** | **Value** | **Rationale** |
| --- | --- | --- | --- | --- |
| b | [1] | Basal TF production rate | 0.2 molecules/h | Near the lowest observable mRNA production rate [1] |
| p_1_ | [2] | Maximal TF1 production rate due to presence of TF1 | 10 molecules/h | Near the high end of mRNA production rates [1] |
| p_2_ | [3,4] | Maximal TF2 production rate due to presence of TF2 | 10 molecules/h | Near the high end of mRNA production rates [1] |
| P_1_ | [2] | Half-maximal copy number for TF1 self-promotion | 10 molecules | With ~20 TF mRNA copies per cell and minimal CY accumulation, mRNA production rate achieves a temporary stable maximum [5]. Therefore, the half-maximal copy number must be ~10 or fewer. |
| P_2_ | [3,4] | Half-maximal copy number for TF2 self-promotion | 10 molecules | Symmetry with P_1_. Early TF production, when CY cannot contribute, is symmetric between TF1 and TF2, so no asymmetry permitted here. |
| h_p_ | [2,3,4] | Hill exponent for TF self-promotion | 2 (unitless) | Hill exponent > 1 required for the switch-like TF self-regulation circuit observed in the absence of CY [5]. Also follows convention of [6]. |
| X_1_ | [7,8] | Half-maximal copy number for TF1 cross-inhibition of TF2 production | 30 molecules | Must be on the same order of magnitude as P_1_ to be relevant to early regulation of TF production. But blocking a promoter of TF1 production diminishes TF1 production more than it enhances TF2 production [5], so X_1_ > P_1_. |
| X_2_ | [9] | Half-max copy number for TF2 cross inhibition of TF1 production | 30 molecules | Symmetry with X_1_. Early TF production, when CY cannot contribute, is symmetric between TF1 and TF2, so no asymmetry permitted here. |
| h_x_ | [7,8,9] | Hill exponent for TF cross-inhibition | 2 (unitless) | Hill exponent > 1 required for the switch-like TF self-regulation circuit observed in the absence of CY [5]. Also follows convention of [6]. |
| s_1_ | [10,11] | Maximal TF1 production rate due to presence of CY1 | 20 molecules/h | CY-driven TF production dominates TF-driven TF production [5], so s_1_ > p_1_. But values higher than this are quite unlikely [1]. |
| s_2_ | [12] | Maximal TF2 production rate due to presence of CY2 | 20 molecules/h | Symmetry with s_1_. |
| S_1_ | [10,11] | Half-maximal concentration of CY1 for promotion of TF1 production | 200 molecules/cell-volume of extracellular space | Cytokine concentration near the surface of Th cells can routinely exceed 1nM = ~100-200 cytokine molecules/cell-volume of extracellular space [13]. S_1_ should be of a similar magnitude if it is a relevant quantity at reasonable CY concentrations. |
| S_2_ | [13] | Half-maximal concentration of CY2 for promotion of TF2 production | 200 molecules/cell-volume of extracellular space | Symmetry with S_1_. |
| h_s_ | [10,11,12] | Hill exponent for CY promotion of TF production | 1 (unitless) | Observing mixed Th effector types in cell culture requires that the dominant positive feedback is gradual rather than switch-like [14]. Also follows convention of [6]. |
| Z_1_ | [15,16] | Half-maximal concentration of CY1 for inhibition of TF2 expression | 2000 or 2400 molecules/cell-volume of extracellular space | CY promotion of TF production is much stronger than CY inhibition of TF production [5], so Z_1_ >> S_1_. |
| Z_2_ | [17,18] | Half-maximal concentration of CY2 for inhibition of TF1 expression | 2000 or 1600 molecules/cell-volume of extracellular space | Symmetry with Z_1_ for analytical model study. Asymmetry for approximating data, because CY2 inhibition of TF1 production is stronger than CY1 inhibition of TF2 production [14], implying Z_2_ < Z_1_. (Mean value held at 2000.) |
| h_z_ | [15,16,17,18] | Hill exponent for CY inhibition of TF production | 1 (unitless) | Follows h_s_ in assuming that CY-mediated feedbacks are gradual rather than switch-like. |
| dTF_1_ | [1] | Degradation rate of TF1 mRNA | 0.15 1/h | Median mRNA half-life is 9 hours [1], but “regulators of cytokine production” have especially short mRNA half-lives, averaging about 4.5 hours [1]. 4.5h half-life = ~0.15/h degradation rate. |
| dTF_2_ | [1] | Degradation rate of TF2 mRNA | 0.15 1/h | Symmetry with dTF_1_. |
| a_1_ | [19] | Maximal production rate of CY1 | 20*3600/(*cvpc*) molecules/cell-volume of extracellular space/h | CY production by individual cells can average 20 molecules secreted per second [20]. This quantity is then converted to the number of molecules secreted into each cell-volume of extracellular space, per hour. See S1 Text for explanation of *cpvc*, which incorporates cell density. |
| a_2_ | [3,21] | Maximal production rate of CY2 | 20*3600/(*cvpc*) molecules/cell-volume of extracellular space/h | Symmetry with a_1_. |
| A_1_ | [19] | Half-maximal copy number of TF1 to promote CY1 production | 80 molecules | TF1 copy number saturates at ~100+ per cell [14], so A_1_ < 100 to be physiologically relevant. But, TF-driven CY expression is invoked long after TF-driven TF expression has reached steady-state rate [5], so A_1_ > P_1_, X_1_. |
| A_2_ | [3,21] | Half-maximal copy number of TF2 to promote CY2 production | 80 molecules | Symmetry with A_1_. |
| h_a_ | [8,19,21] | Hill exponent for TF promotion of CY production | 1 | There is a gradual, rather than switch-like, relationship between TF expression and probability of CY expression [14]. |
| R_1_ | [10,19] | Half-maximal copy number of TF1 to inhibit CY2 production | 80 or 112 molecules | Symmetry with A_1,2_ and for the same rationale as A_1,2_. |
| R_2_ | [3,22] | Half-maximal copy number of TF2 to inhibit CY1 production | 80 or 48 molecules | Symmetry with R_1_ for analytical model study. Asymmetry for approximating data, because CY1 and CY2 production rates are both more impacted by TF2 than by TF1 [14]. This implies that R_2_ < A_1_, and A_2_ < R_1_. (Mean value held at 80.) |
| h_r_ | [3,10,19, 22] | Hill exponent for TF inhibition of CY production | 1 | There is a gradual, rather than switch-like, relationship between TF expression and probability of CY expression [14]. |
| U_1_ | [16,23] | Half-maximal concentration of CY1 to inhibit production of CY2 | 2000 molecules/cell-volume of extracellular space | CY promotion of TF production is much stronger than CY-mediated negative feedbacks [5], so U_1_ >> S_1_. |
| U_2_ | [24,25] | Half-maximal concentration of CY2 to inhibit production of CY1 | 2000 molecules/cell-volume of extracellular space | Symmetry with U_2_. |
| h_u_ | [16,23,24,25] | Hill exponent for CY inhibition of CY production | 1 | Follows h_s_ in assuming that CY-mediated feedbacks are gradual rather than switch-like. |
| dCY_1_ | [1] | Removal rate of CY1 | 0.8/(*cvpc*) + 0.015  1/cell-volume/h | First term implies that most of the cytokine within a 1 cell-volume shell around a cell is consumed within 1 hour. This assumption is validated by the efficacy of cytokine capture assays (e.g. Miltenyi Biotec pt. no. 130-090-479). The second term implies that the half-life of a cytokine protein is ~10x the half-life of an mRNA molecules [1]. See S1 Text for explanation of *cpvc*, which incorporates cell density. |
| dCY_2_ | [1] | Removal rate of CY2 | 0.8/(*cvpc*) + 0.015  1/cell-volume/h | Symmetry with dCY_1_. |
| APC_1_ | [26,27] | Effect of Type-1 APCs on molecular expression dynamics (in terms of an equivalent CY1 concentration) | 0.003 or 0.17 * (98% effective concentration of CY1) | First term is the per-hour probability of an antigen-specific Th cell encountering a dendritic cell, which ranges from 0.003 to 0.17 at biologically realistic dendritic cell frequencies [28]. Second term assumes the influence of a Type-1 APC is as strong as the concentration of CY1 that would stimulate 98% of Th cells to produce TF1. |
| APC_2_ | [26,27] | Effect of Type-2 APCs on molecular expression dynamics (in terms of an equivalent CY2 concentration) | 0.003 or 0.17 * (98% effective concentration of CY2) | Symmetry with APC_1_. |

**REFERENCES**

1. Schwanhausser B, Busse D, Li N, Dittmar G, Schuchhardt J, Wolf J, et al. Global quantification of mammalian gene expression control. Nature. 2011;473(7347):337-42. <https://doi.org/10.1038/nature10098>. PMID: 21593866.
2. Mullen AC, Hutchins AS, High FA, Lee HW, Sykes KJ, Chodosh LA, et al. Hlx is induced by and genetically interacts with T-bet to promote heritable T(H)1 gene induction. Nat Immunol. 2002;3(7):652-8. <https://doi.org/10.1038/ni807>. PMID: 12055627.
3. Jenner RG, Townsend MJ, Jackson I, Sun K, Bouwman RD, Young RD, et al. The transcription factors T-bet and GATA-3 control alternative pathways of T-cell differentiation through a shared set of target genes. Proc Natl Acad Sci U S A. 2009;106(42):17876-81. <https://doi.org/10.1073/pnas.0909357106>. PMID: 19805038.
4. Ouyang W, Lohning M, Gao Z, Assenmacher M, Ranganath S, Radbruch A, et al. Stat6-independent GATA-3 autoactivation directs IL-4-independent Th2 development and commitment. Immunity. 2000;12(1):27-37. <https://doi.org/10.1016/s1074-7613(00)80156-9>. PMID: 10661403.
5. Fang M, Xie H, Dougan SK, Ploegh H, van Oudenaarden A. Stochastic cytokine expression induces mixed T helper cell states. PLoS Biol. 2013;11(7):e1001618. <https://doi.org/10.1371/journal.pbio.1001618>. PMID: 23935453.
6. Yates A, Callard R, Stark J. Combining cytokine signaling with T-bet and GATA-3 regulation in Th1 and Th2 differentiation: a model for cellular decision-making. J. Theor Biol. 2004; 231(2):181-96. <https://doi.org/10.1016/j.jtbi.2004.06.013>. PMID: 15380383.
7. Hwang ES, Szabo SJ, Schwartzberg PL, Glimcher LH. T helper cell fate specified by kinase-mediated interaction of T-bet with GATA-3. Science. 2005;307(5708):430-3. <https://doi.org/10.1126/science.1103336>. PMID: 15662016.
8. Usui T, Preiss JC, Kanno Y, Yao ZJ, Bream JH, O’Shea JJ, et al. T-bet regulates Th1 responses through essential effects on GATA-3 function rather than on IFNG gene acetylation and transcription. J Exp Med. 2006;203(3):755-66. <https://doi.org/10.1084/jem.20052165>. PMID: 16520391.
9. Ouyang W, Ranganath SH, Weindel K, Bhattacharya D, Murphy TL, Sha WC, et al. Inhibition of Th1 development mediated by GATA-3 through an IL-4-independent mechanism. Immunity. 1998;9(5):745-55. <https://doi.org/10.1016/s1074-7613(00)80671-8>. PMID: 9846495.
10. Afkarian M, Sedy JR, Yang J, Jacobson NG, Cereb N, Yang SY, et al. T-bet is a STAT1-induced regulator of IL-12R expression in naïve CD4+ T cells. Nat Immunol. 2002;3(6):549-57. <https://doi.org/10.1038/ni794>. PMID: 12006974.
11. Lighvani AA, Frucht DM, Jankovich D, Yamane H, Aliberti J, Hissong BD, et al. T-bet is rapidly induced by interferon-gamma in lymphoid and myeloid cells. Proc Natl Acad Sci U S A. 2001;98(26):15137-42. <https://doi.org/10.1073/pnas.261570598>. PMID: 11752460.
12. Takeda K, Tanaka T, Shi W, Matsumoto M, Minami M, Kashiwamura S, et al. Essential role of Stat6 in IL-4 signaling. Nature. 1996;380(6575):627-30. <https://doi.org/10.1038/380627a0>. PMID: 8602263.
13. Thurley K, Gerecht D, Friedmann E, Hofer T. Three-dimensional gradients of cytokine signaling between T cells. PLoS Comput Biol. 2015;11(4):e1004206. <https://doi.org/10.1371/journal.pcbi.1004206>. PMID: 25923703.
14. Antebi YE, Reich-Zeliger S, Hart Y, Mayo A, Eizenberg I, Rimer J, et al. Mapping differentiation under mixed culture conditions reveals a tunable continuum of T cell fates. PLoS Biol. 2013;11(7):e1001616. <https://doi.org/10.1371/journal.pbio.1001616>. PMID: 23935451.
15. Albanesi C, Fairchild HR, Madonna S, Scarponi C, De Pita O, Leung DY, et al. IL-4 and IL-13 negatively regulate TNF-alpha- and IFN-gamma-induced beta-defensin expression through STAT-6, suppressor of cytokine signaling (SOCS)-1, and SOCS-3. J Immunol. 2007;179(2):984-92. <https://doi.org/10.4049/jimmunol.179.2.984>. PMID: 17617590.
16. Elser B, Lohoff M, Kock S, Giaisi M, Kirchoff S, Krammer PH, et al. IFN-gamma represses IL-4 expression via IRF-1 and IRF-2. Immunity. 2002;17(6):703-12. <https://doi.org/10.1016/s1074-7613(02)00471-5>. PMID: 12479817.
17. Dickensheets HL, Venkataraman C, Schindler U, Donnelly RP. Interferons inhibit activation of STAT6 by interleukin 4 in human monocytes by inducing SOCS-1 gene expression. Proc Natl Acad Sci U S A. 1999;96(19):10800-5. <https://doi.org/10.1073/pnas.96.19.10800>. PMID: 10485906.
18. Losman JA, Chen XP, Hilton D, Rothman P. Cutting edge: SOCS-1 is a potent inhibitor of IL-4 signal transduction. J Immunol. 1999;162(7):3770-4. PMID: 10201892.
19. Djuretic IM, Levanon D, Negreanu V, Groner Y, Rao A, Ansel KM. Transcription factors T-bet and Runx3 cooperate to activate Ifng and silence Il4 in T helper type 1 cells. Nat Immunol. 2007;8(2):145-53. <https://doi.org/10.1038/ni1424>. PMID: 17195845.
20. Han Q, Bradshaw EM, Nilsson B, Hafler DA, Love JC. Multidimensional analysis of the frequencies and rates of cytokine secretion from single cells by quantitative microengraving. Lab Chip. 2010;10(11):1391-400. <https://doi.org/10.1039/b926849a>. PMID: 20376398.
21. Tykocinski LO, Hajkova P, Chang HD, Stamm T, Sozeri O, Lohning M, et al. A critical control element for interleukin-4 memory expression in T helper lymphocytes. J Biol Chem. 2005;280(31):28177-85. <https://doi.org/10.1074/jbc.M502038200>. PMID: 15941711.
22. Chang S, Aune TM. Dynamic changes in histone-methylation ‘marks’ across the locus encoding interferon-gamma during the differentiation of T helper type 2 cells. Nat Immunol. 2007;8(7):723-31. <https://doi.org/10.1038/ni1473>. PMID: 17546034.
23. Venkataraman C, Leung S, Salvekar A, Mano H, Schindler U. Repression of IL-4-induced gene expression by IFN-gamma requires Stat1 activation. J Immunol. 1999;162(7):4053-61. PMID: 10201928.
24. Knosp CA, Johnston JA. Regulation of CD4+ T-cell polarization by suppressor of cytokine signaling proteins. Immunology. 2012;135(2):101-11. <https://doi.org/10.1111/j.1365-2567.2011.03520.x>. PMID: 22044277.
25. Seki Y, Inoue H, Nagata N, Hayashi K, Fukuyama S, Matsumoto K, et al. SOCS-3 regulates onset and maintenance of T(H)2-mediated allergic responses. Nat Med. 2003;9(8):1047-54. <https://doi.org/10.1038/nm896>. PMID: 12847520.
26. Moser M. Regulation of Th1/Th2 development by antigen-presenting cells in vivo. Immunobiology. 2001;204(5):551-7. <https://doi.org/10.1078/0171-2985-00092>. PMID: 11846218.
27. Sallusto F, Lanzavecchia A. The instructive role of dendritic cells on T-cell responses. Arthritis Res. 2002;4Suppl3:S127-32. <https://doi.org/10.1186/ar567>. PMID: 12110131.
28. Celli S, Day M, Muller AJ, Molina-Paris C, Lythe G, Bousso P. How many dendritic cells are required to initiate a T-cell response? Blood. 2012;120(19):3945-8. <https://doi.org/10.1182/blood-2012-01-408260>. PMID: 22995897.
