# Supplementary figures and images for: Quorum sensing via dynamic cytokine signaling comprehensively explains divergent patterns of effector choice among helper T cells
Source: PLoS Comput Biol. 2020 Jul 30;16(7):e1008051. doi: 10.1371/journal.pcbi.1008051 (PMC7392205; doi:10.1371/journal.pcbi.1008051)

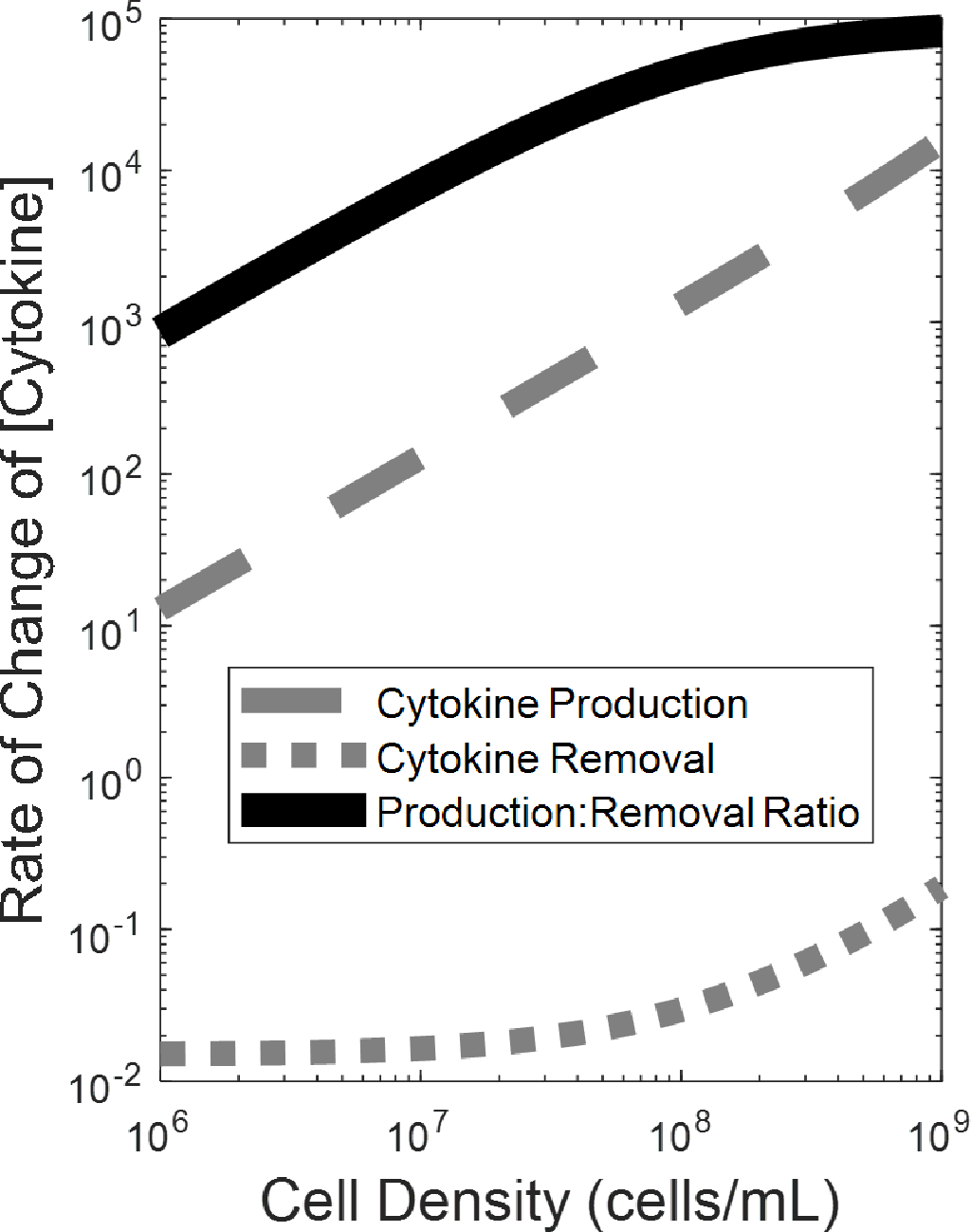

Supplement: S1 Fig — However, production increases faster than removal. Therefore, the ratio of production rate: removal rate increases with cell density. (TIF) [file pcbi.1008051.s006.tif]

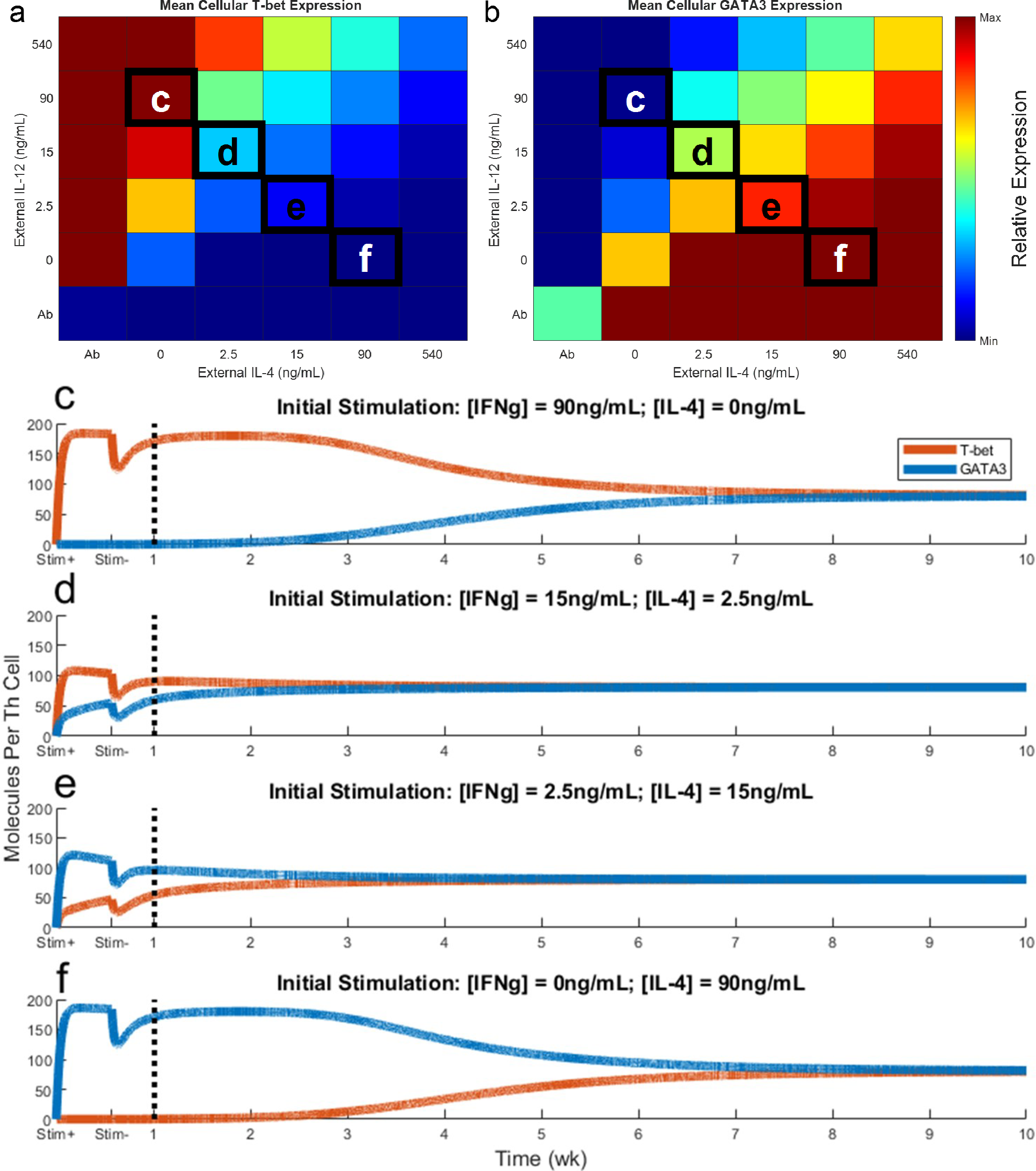

Supplement: S2 Fig — Experiments and model were both run at 2*106 cells/mL. (a) T-bet and (b) GATA3 expression, after 1 week in culture, both follow a continuum based on exogeneous stimulation (or blocking via antibodies), just as observed in [33] (c-f) Regardless of the input stimulation and the transient effector balance achieved at 1 week, all conditions converge on a common mixed effector type after approximately 10 weeks. (TIF) [file pcbi.1008051.s007.tif]

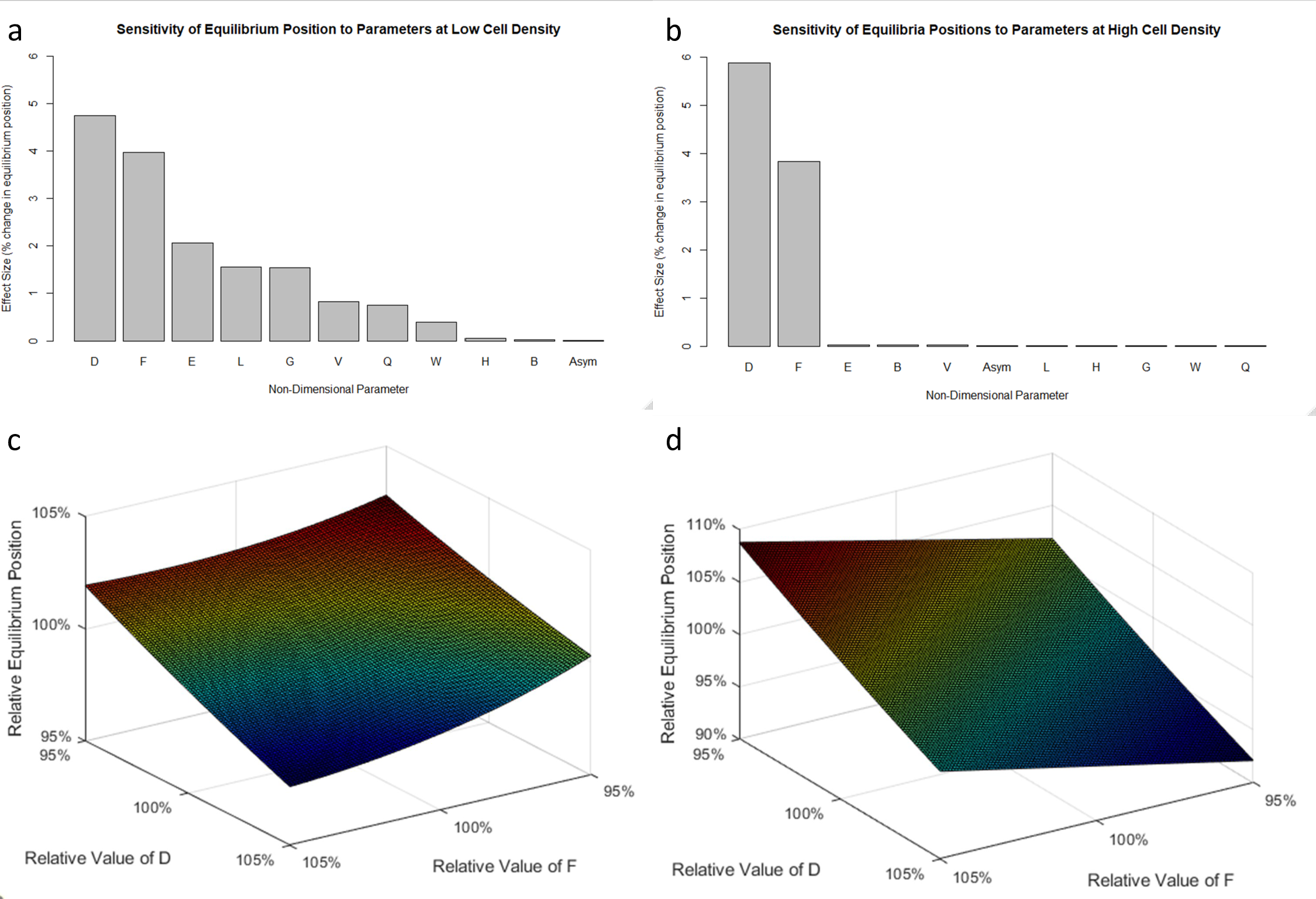

Supplement: S3 Fig — “Asym” can be shown analytically to have no effect on equilibrium position, and therefore marks an effect size that must be insignificant. (a) Near the low-density point-of-interest, the net effect size on equilibrium position of each parameter, controlling for variation in all other parameters, does not exceed 5%. (b) Near the high-density point-of-interest, the net effect size on equilibria position of each parameter, controlling for variation in all other parameters, does not exceed 6%. (c) Near the low-density point-of-interest, the most influential parameters (D and F) exhibit smooth, slight, largely linear, and largely non-interacting effects on the position of the equilibrium. (d) Near the high-density point-of-interest, these parameters still exhibit smooth, slight, largely linear, and largely non-interacting effects on the position of the equilibria. (TIF) [file pcbi.1008051.s008.tif]

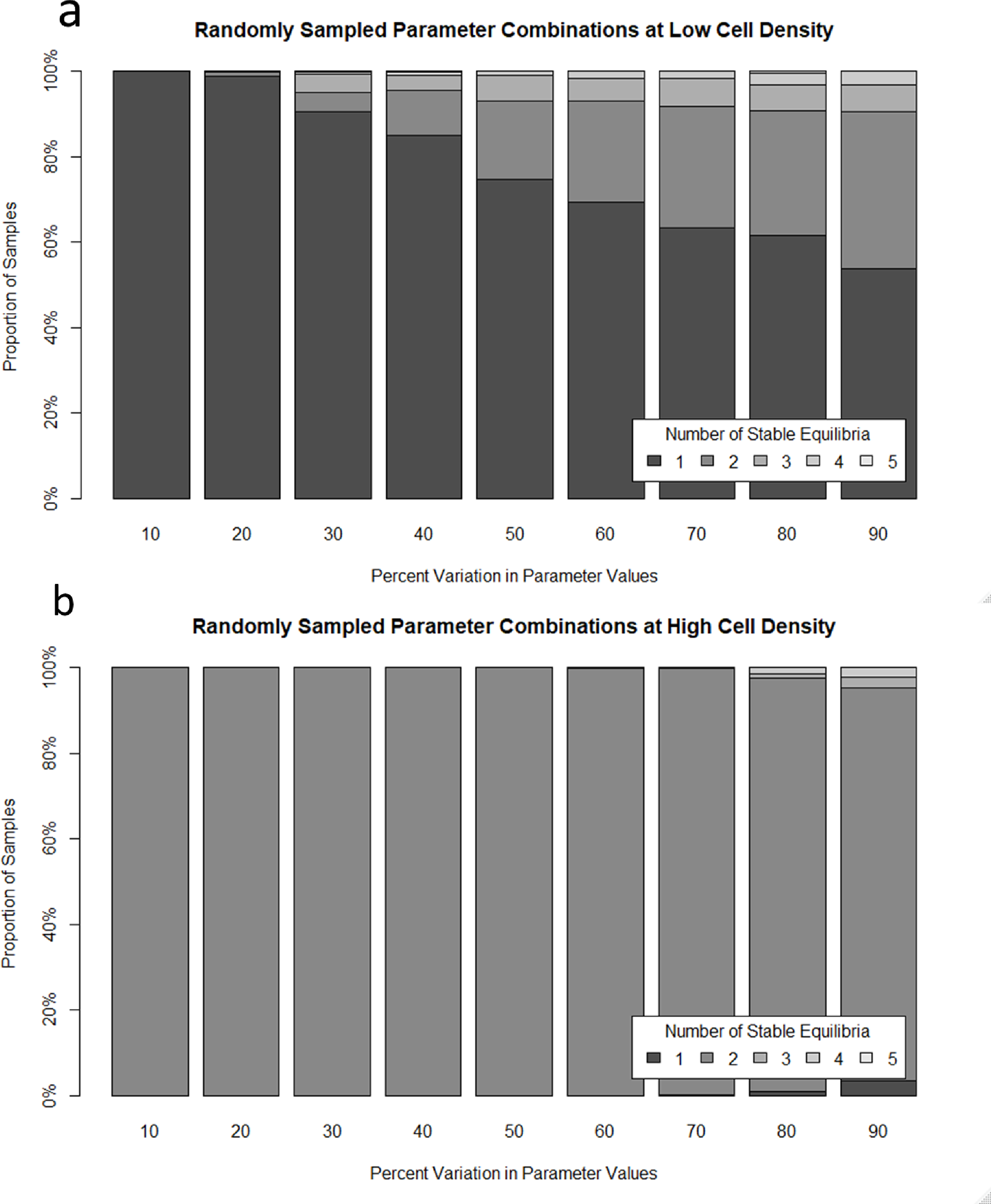

Supplement: S4 Fig — (a) Straying from the low-density point-of-interest, new regimes of model behavior (i.e. new numbers of stable equilibria) appear with as little as 20% variation in parameter values, but over half of sampled parameter sets still follow the original model behavior. (b) Straying from the high-density point-of-interest, new regimes of model behavior do not appear even up to 50% variation in parameter values. (TIF) [file pcbi.1008051.s009.tif]
