# Supplementary material for: Quorum sensing via dynamic cytokine signaling comprehensively explains divergent patterns of effector choice among helper T cells
Source: PLoS Comput Biol. 2020 Jul 30;16(7):e1008051. doi: 10.1371/journal.pcbi.1008051 (PMC7392205; doi:10.1371/journal.pcbi.1008051)
Supplement: Attachment — Submitted filename: plos review.docx [file pcbi.1008051.s010.docx]

In this paper, authors apply techniques of understanding swarm behavior in ecology to a decision-making problem in the adaptive immune system and specifically on effector choice among Th cells. They mainly focus on explaining the reasons for polarized group effector decisions in vivo and mixed effector types in vitro. To answer this question, they design a model that consists of four ordinary differential equations with state variables transcription factors and cytokines that each self-activates itself or cross inhibit others. Then they generalized their model to the stochastic differential equation (SDE) since not only the mean but the whole distribution of molecular expression across a group of Th cells matter. Finally, they explain the importance of dynamic signaling in the presence of antigen-presenting cells (APCs) and the advantages that dynamic signaling could provide. They answer this question by slight modification of their model and adding the role APC into cytokine equations.

I found this paper very interesting and the results are new. The questions and analysis are clear and answered comprehensively. I think it is really good work and fit to be published in PLOS **Computational Biology.**

I have a few questions and comments:

Can you modify your model to include Th17 cells?

Line 607: In the caption, please explain Figure 1 in more detail. It is hard to understand by just looking at the model schematic.

Line 684: In Figure 7, distinguish which cells are APC, Th1, Th2, and Th1-Th1 hybrids.

It would be great if the authors allocate a section in the supplementary materials for the development of their ODE model to SDE and explain the analysis.

The reason for using the stochastic model in your paper is convincing but I think that SDE demands some assumption that biologically does not make sense? In SDE, one makes the state parameters stochastic by assigning a variation to it using a Wiener process while other parameters are kept deterministic. For example, in your SDE model, you treated every parameter as a fixed value, while assigning random normally distributed noise to state variables. But it seems that the uncertainty in the values of the state parameters should be inherited from the uncertainty in the parameter values (e.g. “b” as Basal TF production rate) because state parameters are functions of other parameters.
