# Supplementary material for: Quorum sensing via dynamic cytokine signaling comprehensively explains divergent patterns of effector choice among helper T cells
Source: PLoS Comput Biol. 2020 Jul 30;16(7):e1008051. doi: 10.1371/journal.pcbi.1008051 (PMC7392205; doi:10.1371/journal.pcbi.1008051)
Supplement: Attachment — Submitted filename: Schrom_ResponseToReviewers_v7.docx [file pcbi.1008051.s011.docx]

Dear PLoS Computational Biology Editors,

Thank you for your positive response to our manuscript! Please find below the editor and reviewer comments in red, along with our responses and pointers to the text where the requested changes have been made in black. Please note that Line Numbers refer to the marked-up copy of our manuscript, as the clean copy of the manuscript has been pulled apart into separate documents (i.e. each Supplemental Text is now its own file, as are the Supplemental Figure Legends, Table, and References).

Sincerely,

Edward Schrom, on behalf of all authors

**Editor Comments**

“*First, sorry for the time it has taken for the reviews. I agree with both reviewers that the paper is very interesting and with minor modifications will be a very nice publication in PLOS computational biology*”

**Response:** Thank you! We very much appreciate your positive view of our work, and we hope you find that our modifications have improved it further.

**Reviewer #1 Comments**

*“In this paper, authors apply techniques of understanding swarm behavior in ecology to a decision-making problem in the adaptive immune system and specifically on effector choice among Th cells. They mainly focus on explaining the reasons for polarized group effector decisions in vivo and mixed effector types in vitro. To answer this question, they design a model that consists of four ordinary differential equations with state variables transcription factors and cytokines that each self-activates itself or cross inhibit others. Then they generalized their model to the stochastic differential equation (SDE) since not only the mean but the whole distribution of molecular expression across a group of Th cells matter. Finally, they explain the importance of dynamic signaling in the presence of antigen-presenting cells (APCs) and the advantages that dynamic signaling could provide. They answer this question by slight modification of their model and adding the role APC into cytokine equations.”*

**Response:** This is an excellent summary of our work! We are glad to know that the structure and aims of our manuscript were so clear.

*“I found this paper very interesting and the results are new. The questions and analysis are clear and answered comprehensively. I think it is really good work and fit to be published in PLOS* ***Computational Biology.”***

**Response:** Thank you for your appreciation of our work!

*“I have a few questions and comments: Can you modify your model to include Th17 cells?”*

**Response:** Modification of our model to incorporate Th17, Th9, iTreg, or other Th effector types is certainly possible. Our model structure relies on the simplifying assumption that each Th effector type is controlled by a single master transcription factor (here, T-bet for Th1 and GATA3 for Th2) and has a single master cytokine (here, IFNγ for Th1 and IL-4 for Th2). Similar assumptions are routinely made for other effector types (e.g. RORγt as the master transcription factor and IL-21 as the master cytokine for Th17). For example, Martinez-Sanchez et al (PLOS Comp Biol, 2015) and van den Ham and de Boer (Int Immunol, 2008) have both insightfully used this “master regulator” assumption to mathematically recover experimental observations of Th differentiation into effector types other than Th1 and Th2. Thus, adding or substituting pairs of equations into our model to represent transcription factor and cytokine expression for Th17 – or any other Th effector type – is certainly justifiable. In fact, molecular interactions mirroring those studied here also govern differentiation into Th17 vs. iTreg effector types (Zhou, Chong, and Littman. Immunity. 2009). One may be able to adapt this 4-equation model to instead represent Th17 vs. iTreg differentiation and obtain similar results.

Nonetheless, we restricted our model to Th1 and Th2 effector types for several reasons. First, the construction of our model required an exhaustive search of the literature to catalogue all discovered biochemical interactions among the master Th1 and Th2 transcription factors and cytokines. This search was most likely to be fully comprehensive for Th1 and Th2, because these effector types have been known and studied the longest. Second, the types of data we wanted to compare to our model predictions were most readily available from experiments focused on Th1 vs. Th2 differentiation (Fig. 2, Fig. 3, and Supp. Fig. 2). For these reasons, we made Th1 vs. Th2 differentiation the basis of our model. Nor did we add further pairs of equations onto this basis, because performing and interpreting mathematical analyses such as those explained in Supp. Text 4 become increasingly difficult as the number of equations in the model grows. And, while we do not anticipate that the general results of this study would change with the inclusion of more Th effector types, Th quorum sensing and its potential adaptive benefits are easiest to describe and depict graphically for just two effector types.

Still, we agree that the relation between our model and other effector types is an interesting and pertinent question that ought to be addressed in the main text, so we have added a new paragraph (Lines 395-402) to the Discussion. We believe that adapting this model to address more specific questions regarding how quorum sensing impacts Th17 differentiation across contexts would make an excellent follow-up study.

*“Line 607: In the caption, please explain Figure 1 in more detail. It is hard to understand by just looking at the model schematic.”*

**Response:** We have added several sentences to the caption of Figure 1 to explain what the four state variables of our model are, what their spatial scales are, and how their interactions with each other are depicted (Lines 650-4).

*“Line 684: In Figure 7, distinguish which cells are APC, Th1, Th2, and Th1-Th1 hybrids.”*

**Response:** Good catch! We have now done so in the Figure 7 caption (Lines 731-2).

*“It would be great if the authors allocate a section in the supplementary materials for the development of their ODE model to SDE and explain the analysis.”*

**Response:** We have now inserted Supp. Text 3 (Lines 846-73), which gives the full set of SDE equations, justifies the mathematical formulation of the stochastic terms, tells how these stochastic terms should be interpreted biologically, and explains how simulated sample paths of the SDEs were used in analysis.

*“The reason for using the stochastic model in your paper is convincing but I think that SDE demands some assumption that biologically does not make sense? In SDE, one makes the state parameters stochastic by assigning a variation to it using a Wiener process while other parameters are kept deterministic. For example, in your SDE model, you treated every parameter as a fixed value, while assigning random normally distributed noise to state variables. But it seems that the uncertainty in the values of the state parameters should be inherited from the uncertainty in the parameter values (e.g. “b” as Basal TF production rate) because state parameters are functions of other parameters.”*

**Response:** There are multiple ways to construct a stochastic differential equation. You are certainly correct that one way is to add normally distributed noise to specific parameters using independent Wiener processes. A second way is to simply add a noise term involving a single Wiener process after the deterministic part of the equation. The latter method seems most appropriate to us, for two reasons: biological accuracy and mathematical convenience.

To first address biological accuracy, we know that all four state variable molecules – T-bet, GATA3, IFNγ, and IL-4 – appear to follow lognormal expression distribution across cells in experiments (Fang et al. PLOS Biol. 2013.; Han et al. Lab Chip. 2010). Accordingly, we used the geometric Brownian motion noise term (noise coefficient)*(state variable)*dW, which creates lognormal stochasticity in the state variable, just as observed experimentally. The downside to this approach is that the noise coefficient effectively integrates over all sources of fluctuations in the state variable, such that noise cannot be attributed to any particular biochemical parameter. The benefit to this approach is that the distributions at the level of the state variables are biologically realistic. Because we are interested in the consequences of cell-to-cell variability, rather than the biochemical source of cell-to-cell variability, this implementation seems appropriate to us.

On the other hand, introducing noise to specific biochemical parameters may not be biologically realistic, because it assumes that the cell-to-cell variability in those parameters is normally distributed. We do not know if this is true. Nor do we know whether normally distributed noise in several biochemical parameters would propagate to ultimately create lognormally distributed cell-to-cell variability at the level of the state variable, as desired. Nor do we have a sound basis for choosing which biochemical parameters should have noise added to them, and which should not. So it seems this method of introducing noise risks greater biological inaccuracy.

Secondly, our introduction of a term like +$\left[ n_{TF}\boldsymbol{TF}_{\boldsymbol{1}} \right]{dW}_{TF1}$ to each equation is mathematically concise. On the other hand, if noise is added to individual parameters, each noisy parameter receives its own independent Wiener process, whose standard deviation is the entire coefficient of that parameter from the deterministic portion of the equation. These independent Wiener processes can then be condensed into single Wiener process whose standard deviation is the square root of the sum of squares of the individual standard deviations. In practice, taking our equation for TF1 as an example, if we only added noise to “b” and to “s_1_,” this would result in the introduction of a noise term like:

$$n\sqrt{\left( \frac{X_{2}^{h_{x}}}{X_{2}^{h_{x}}+\boldsymbol{TF}_{2}^{h_{x}}} \right)^{2}+\left( \left( \frac{{\boldsymbol{CY}_{1}}^{h_{s}}}{S_{1}^{h_{s}}+{\boldsymbol{CY}_{1}}^{h_{s}}} \right)\left( \frac{Z_{2}^{h_{z}}}{Z_{2}^{h_{z}}+\boldsymbol{CY}_{2}^{h_{z}}} \right) \right)^{2}}{dW}_{TF1}$$

Of course, we might like to add noise to other biochemical parameters, too, which would quickly become unwieldy.

All this is to say that we believe our chosen method of constructing SDEs is most appropriate for our purposes. Nonetheless, we appreciate your point that our method might cause confusion over how to interpret the noise coefficient “n.” Thus, we have explained in our new Supp. Text 3 that the noise coefficient is a quantification of the total cell-to-cell variability of a state variable, but it cannot be interpreted as stochastic fluctuations of any biochemical parameter in particular (Lines 849-52). Moreover, we have been careful throughout the final section of results and the discussion to refer to cell-to-cell variability in total cytokine “expression” rather than in “secretion,” to prevent readers from erroneously inferring that we are specifically introducing noise into the cytokine secretion rate parameter “a,” or any other biochemical parameter.

**Reviewer #2 Comments:**

*“This is an excellent article that uses a simple model to illustrate how T helper cells make collective choices without central control by using quorum sensing approaches. It is an elegant explanation of differences observed in vivo (heterogenous mixtures of Th1 and Th2) vs polarized choices in vitro driven by a bifurcation in dynamics. The model and analysis explain the role of APC instruction, cytokine signaling and cell density in determining Th cell fate. This work has potential to fundamentally change understanding of effector choice, and perhaps spur other similar investigations into “swarm”-like mechanisms in immune response more generally.”*

**Response:** Thank you so much for your enthusiasm for our work!

*“The introduction is concise and compelling and nicely motivates the questions to be addressed in the paper. I suggest including an explanation of quorum sensing which may not be obvious to all readers.”*

**Response:** Thanks! We have now provided a more clear definition of what quorum sensing is on Lines 57-9.

*“The figures are very clear – starting with Fig 1 that summarizes the dynamics of the model through the last figure that illustrates the what the model means for Th cell choice.”*

**Response:** Thanks! We are glad the figures clearly convey the important points of the manuscript.

*“In the pp starting on line 230, I found the cross scale vs within scale terminology confusing. Why not refer to this as within cell vs between cells?”*

**Response:** This distinction is indeed hard to discuss clearly! In our model, molecules exist at two scales, which match the names you’ve suggested:

1) transcription factors are “within cells.”

2) cytokines are “between cells.”

However, in the Results, we are not categorizing molecules based on the scales at which they exist. Instead, we are categorizing processes based on whether they involve just one of the “within-cell” and “between-cell” scales, or both of them:

1) processes by which transcription factors affect each other’s expression OR by which cytokines affect each other’s expression are “within-scale,” because they are inherently confined to a single scale, whichever scale that is.

2) processes by which transcription factors affect cytokine expression OR vice-versa are “cross-scale,” because they inherently involve both scales.

We hope that clarifies our terminology for you! To preemptively clarify this for readers, we have incorporated this explanation of the terminology into the Results at Lines 237-43.

*“There are several relevant papers to consider citing that describe lymphocytes as a swarm, quorum, and by analogy to ants.
- Switching between individual and collective motility in B lymphocytes is controlled by cell-matrix adhesion and inter-cellular interactions (Theraluz
- Quorum Regulation via Nested Antagonistic Feedback Circuits Mediated by the (Zenke S et al., Immunity, 2020)
- Distributed Adaptive Search in T Cells: Lessons From Ants (Moses et al Fronteirs in Immunology 2019)”*

**Response:** References to these papers are excellent additions to our introduction and discussion, and we have now incorporated them at Lines 65, 71, 119-120, and 385.
